# Assessment of flux through oleoresin biosynthesis in epithelial cells of loblolly pine resin ducts

**DOI:** 10.1093/jxb/ery338

**Published:** 2018-10-11

**Authors:** Glenn W Turner, Amber N Parrish, Jordan J Zager, Justin T Fischedick, B Markus Lange

**Affiliations:** 1 Institute of Biological Chemistry and M.J. Murdock Metabolomics Laboratory, Washington State University, Pullman, WA, USA; 2 Pure Analytics, Santa Rosa, CA, USA

**Keywords:** Diterpene resin acid, gas chromatography–mass spectrometry, genome-scale model, metabolic flux, monoterpene, oleoresin, resin duct, secretory cell type, sesquiterpene, transcriptome

## Abstract

The shoot system of pines contains abundant resin ducts, which harbor oleoresins that play important roles in constitutive and inducible defenses. In a pilot study, we assessed the chemical diversity of oleoresins obtained from mature tissues of loblolly pine trees (*Pinus taeda* L.). Building on these data sets, we designed experiments to assess oleoresin biosynthesis in needles of 2-year-old saplings. Comparative transcriptome analyses of single cell types indicated that genes involved in the biosynthesis of oleoresins are significantly enriched in isolated epithelial cells of resin ducts, compared with those expressed in mesophyll cells. Simulations using newly developed genome-scale models of epithelial and mesophyll cells, which incorporate our data on oleoresin yield and composition as well as gene expression patterns, predicted that heterotrophic metabolism in epithelial cells involves enhanced levels of oxidative phosphorylation and fermentation (providing redox and energy equivalents). Furthermore, flux was predicted to be more evenly distributed across the metabolic network of mesophyll cells, which, in contrast to epithelial cells, do not synthesize high levels of specialized metabolites. Our findings provide novel insights into the remarkable specialization of metabolism in epithelial cells.

## Teaser

Genome-scale models capture the remarkable specialization of epithelial cells for oleoresin biosynthesis in needles of loblolly pine (*Pinus taeda* L.).

## Introduction

**Fig. 1.**
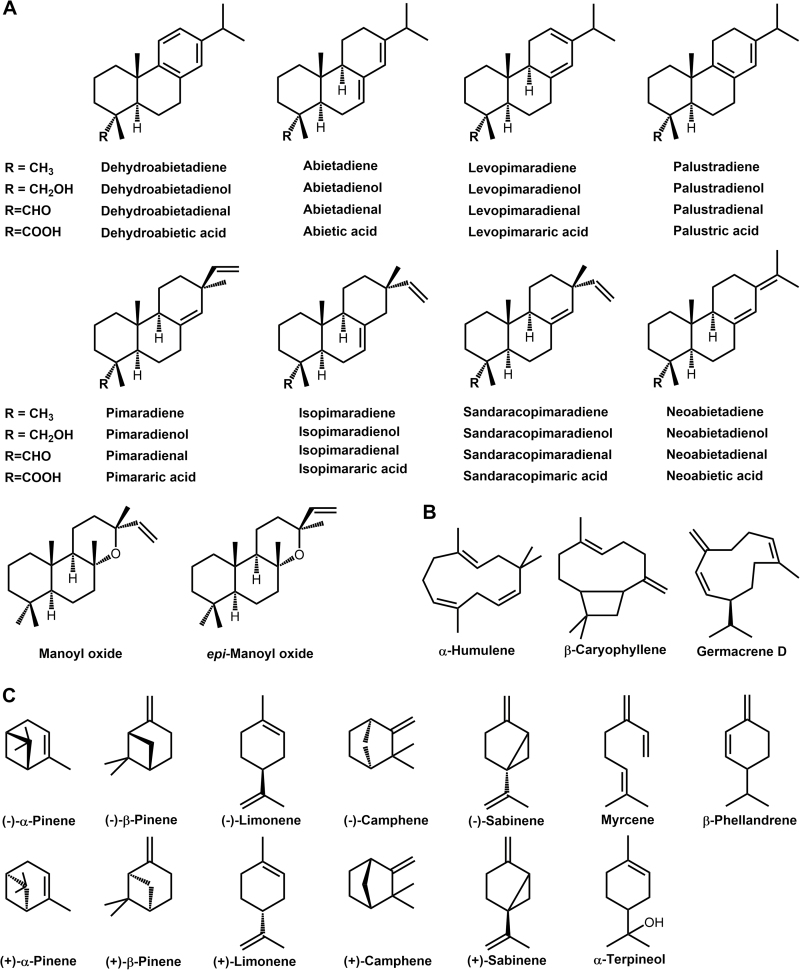
Major loblolly pine oleoresin constituents. Chemical structures of (A) diterpenoids, (B) sesquiterpenes, and (C) monoterpenes identified in this study.

Oleoresins form constitutively and abundantly in the stems and needles of many conifers. They can also be synthesized and secreted as an induced defensive response to herbivore or pathogen attack (Franceschi *et al.*, 2005; Keeling and Bohlmann, 2006). The stems of the genera *Abies*, *Cedrus*, *Tsuga*, and *Pseudolarix* accumulate oleoresins constitutively in sac-like structures called resin blisters, whereas *Pinus*, *Picea*, *Larix*, and *Pseudotsuga* resins are found in tubular networks of ducts (Fahn, 1988). Wounding and methyl jasmonate treatment can elicit the formation of resin ducts even in conifer species that normally lack ducts (Hudgins and Franceschi, 2004). The chemistry of conifer oleoresins is characterized by mixtures of volatiles (primarily monoterpenes) and semi-volatiles (primarily diterpenoids) (Joye and Lawrence, 1967; Norin, 1972) (Fig. 1).

The naval stores industry has a long history of converting terpenoid oleoresins into rosin and turpentine, which continue to have commercial uses as adhesives, inks, emulsifiers, solvents, fragrances, and resins (da Silva Rodrigues-Corrêa *et al.*, 2012). With the adoption of less expensive, petroleum-derived substitutes, beginning in the 1960s, the commercial importance of oleoresins steadily decreased. However, oleoresins, which are characterized by a high volumetric energy density and high degree of reduction, might be poised for a comeback in economies that employ green biofuels and bioproducts from non-food feedstocks (Vallinayagam *et al.*, 2014).

During the past few years, the field of conifer biochemistry and genetics has witnessed exciting progress, and loblolly pine (*Pinus taeda* L.) has emerged as a model system. A draft reference sequence for the ~22 Gb loblolly pine genome (approximately seven times the size of the human genome) was recently completed (Neale *et al.*, 2014). Association genetics studies have identified single nucleotide polymorphisms correlating with location and environment (Eckert *et al.*, 2010), primary metabolite profiles (Eckert *et al.*, 2013), drought tolerance (González-Martínez *et al.*, 2006), wood characteristics (Palle et al., 2011, 2013), and oleoresin accumulation (Westbrook *et al.*, 2013, 2015). Many of the biosynthetic genes involved in the biosynthesis of monoterpenes and diterpene resin acids, the major constituents of oleoresins of loblolly pine and closely related members of the genus *Pinus*, have been cloned and functionally characterized (Phillips *et al.*, 2003; Ro *et al.*, 2005; Ro and Bohlmann, 2006; Geisler *et al.*, 2016).

Although progress has been made relating to the cell biology and enzymology of glandular trichome cells, more significant gaps remain in our knowledge concerning many aspects of the biology of resin ducts. Here, we integrate cell-type-specific transcriptome data and genome-scale mathematical models to compare flux distribution in epithelial cells of resin ducts and neighboring mesophyll cells of needles. Our analyses indicate that there are commonalities among the metabolic networks of secretory cell types, including those present in resin ducts and glandular trichomes.

## Materials and methods

### Plant growth and harvest

The collection of loblolly pine trees (*Pinus taeda* L.) for the analytical pilot study was grown in a greenhouse under ambient lighting, with supplementary heating to 15 °C on cold days (with no other direct temperature control). Three random trees were harvested at approximately 4 years of age. Bare root seedlings for the main study (Atlantic Coastal provenance; used as tissue source for oleoresin and transcriptome analyses) were obtained from Plum Creek Nursery (now part of Weyerhaeuser Inc.) and maintained under greenhouse conditions [illumination 16 h day, 8 h night (250–500 µmol m^−2^ s^−1^); temperature 24 °C day, 20 °C night; relative humidity 45–55%]. Plants were harvested at approximately 2 years of age.

### Analysis of semi-volatiles

To allow the analysis of potential compositional differences between tissue types, bark (cortex and phloem) was peeled away from secondary xylem of the branches and main stem (for details see Supplementary Fig. S1 at *JXB* online). Tissue samples were homogenized using a Model No. 1 Wiley Mill (Thomas Scientific, Philadelphia, PA, USA) under cooling with liquid nitrogen. The frozen homogenate was placed in a lyophilizer (FreeZone 6L, Labconco, Kansas City, MO, USA) for 48 h and then stored at –20 °C until further processing (maximum storage time 6 d). Aliquots of frozen tissue homogenate (50 mg dry weight) were placed in a cellulose extraction thimble (10 × 50 mm; Whatman, Maidstone, UK). Samples were extracted using 15 ml acetone containing 10 µl ml^–1^ tricosanoic acid as internal standard to assess the extraction efficiency (Sigma-Aldrich, St. Louis, MO, USA) by percolating the mixture at 5–10 cycles h^–1^ for 6 h. The extract was then stored in glass test tubes at –20 °C until further processing (storage time up to 6 d). The acetone extract was evaporated to dryness (EZ-Bio, GeneVac Ltd, Ipswich, UK), the residue was dissolved in 5 ml ethyl acetate, and 1 ml of the supernatant was transferred into a 2 ml glass vial. The solvent was removed (EZ-Bio, GeneVac Ltd, Ipswich, UK) and the remainder was subjected to derivatization by adding 200 µl methanol and 200 µl trimethylsilyldiazomethane (2 M in diethyl ether; Sigma-Aldrich, St. Louis, MO, USA) and maintaining the mixture at 23 °C for 20 min in a vial with a polytetrafluoroethylene-lined cap. Samples were dried under a steady stream of nitrogen gas and the residue was resuspended in 200 µl ethyl acetate containing 0.1 mg ml^–1^ eicosane as external standard to determine the injection reliability.

Extracts were analyzed by gas chromatography with flame ionization detection (GC-FID) using the following conditions: model 6890N GC (Agilent Technologies, Santa Clara, CA, USA), HP-5 column (30 m×0.25 mm; 0.25 µM film thickness; Agilent Technologies, Santa Clara, CA, USA), helium as carrier gas at 1 ml min^–1^, inlet temperature 270 °C (splitless mode), injection volume of 1 µl, detector temperature 280 °C, and an oven temperature gradient of 210 to 280 °C at 2.5 °C min^–1^, with a 5 min hold at the final temperature, before transitioning back to the initial conditions. Quantitation was performed by using ChemStation B.03.02 software (Agilent Technologies, Santa Clara, CA, USA) based upon calibration curves with known amounts of authentic standards and normalization to the sample weight and peak area of the internal standard. Alternatively, extracts were analyzed by gas chromatography with single quadrupole mass spectrometric detection (GC-MS) using the following conditions: 6890N GC interfaced with 5973 Inert MS (Agilent Technologies, Santa Clara, CA, USA), which was operated under the conditions developed by Adams (2007). Metabolites were identified on the basis of chromatographic and spectral comparisons against the NIST 05 library (https://www.nist.gov. Last accessed on October 01, 2018.), the Adams essential oil library (http://essentialoilcomponentsbygcms.com/. Last accessed on October 01, 2018.), and a comprehensive in-house spectral library generated with authentic standards. Analytes were quantified on the basis of calibration curves obtained with known amounts of authentic standards.

### Analysis of volatiles

For the pilot study (loblolly pine tree samples), aliquots of frozen tissue homogenate [~10 g fresh weight (FW)] were extracted by hydrodistillation using a condenser-cooled Likens–Nickerson apparatus [50 ml water and 10 µl of isobutyl benzene (internal standard) with sample in one flask and 10 ml n-hexane in separate flask] (Ringer *et al.*, 2003). For the main study (needles from saplings), these amounts were scaled down to approximately 5 g FW extracted using 50 ml water with 5 µl internal standard and 5 ml n-hexane. For the pilot study, constituents were identified by GC-FID (6890N; Agilent Technologies, Santa Clara, CA, USA), which was operated with a DB-WAX column (60 m×0.25 mm×0.25 µm; J&W Scientific, Santa Clara, CA, USA) and the following settings: front inlet and detector temperature 270 °C, inlet mode splitless, injection volume 1 µl, carrier gas (helium) flow 0.9 ml min^–1^; initial oven temperature 85 °C (held for 5 min), then a linear gradient to 130 °C at 10 °C min^–1^ (held for 2 min), a second gradient to 150 °C at 4 °C min^–1^ (held for 12 min), and a third gradient to 220 °C at 50 °C min^--–1^ (held for 10 min). For the main study (loblolly pine saplings), the oven temperature program was as follows: initial temperature 85 °C (held for 4 min), then a linear gradient to 130 °C at 3 °C min^–1^ (held for 15 min), and a second gradient to 235 °C at 8 °C min^–1^ (held for 12 min). Quantitation was achieved by using ChemStation B.03.02 software (Agilent Technologies, Santa Clara, CA, USA) based upon calibration curves with known amounts of authentic standards and normalization to the sample weight and peak area of the internal standard. Chiral separations, on a separate GC-FID instrument, were performed as previously described (Srividya *et al.*, 2015, 2016) and metabolites were identified by comparison with the chromatographic retention characteristics of authentic standards. As a third alternative, analytes were identified by GC-MS (6890N GC interfaced with 5973 Inert MS; Agilent Technologies, Santa Clara, CA, USA), which was operated under the conditions developed by Adams (2007). Metabolites were identified on the basis of chromatographic and spectral comparisons as described above.

### Tissue preparation for microscopy

Developing and mature fasicles were dissected and treated overnight with a fixative containing 4% (v/v) glutaraldehyde in 50 mM PIPES buffer (pH 7.2). Samples were post-fixed in aqueous 0.5% (w/v) OsO_4_ for 2 h at 20 °C and then dehydrated in an ethanol series. The ethanol was then exchanged for propylene oxide, with subsequent infiltration of Spurr’s resin (Ted Pella, Redding, CA, USA). Thick sections (1 µm) were cut with glass knives using an Ultracut R microtome (Leica Microsystems, Buffalo Grove, IL, USA) and stained with toluidine blue. Sections were then photographed with a BH-2 Light Microscope (Olympus America Inc., Center Valley, PA, USA).

### Laser microdissection of epithelial and mesophyll cells, and subsequent transcriptome analyses

Separate pools of needles were harvested from three different saplings, shock-frozen in liquid nitrogen, and stored at –80 °C until further use. Frozen needles were cut into segments of approximately 1 cm length. Four to ten segments were then placed in a microfuge tube. RNAse-free water was added and the samples were shock-frozen with liquid nitrogen. The plastic end of the microfuge tube was cut off and the ice cone containing needle segments was forced out with the help of a small metal rod. The ice was then glued to a cryostat specimen holder with frozen Optimum Cutting Temperature formulation according to the manufacturer’s instructions (Tissue-Tek® from Sakura Finetek, Torrance, CA, USA). Sections were cut (roughly 10 µm thickness) with a disposable cryostat knife, which had been surface-cleaned with RNase Away (Thermo Fisher Scientific, Waltham, MA, USA), using a CM 3050 Cryostat (Leica Microsystems, Wetzlar, Germany). Sections were placed on RNase-free membrane-coated PEN slides (Carl Zeiss Microscopy, Thornwood, NY, USA) and immediately placed on a pre-cooled metal tray within the cryostat. Samples were then transferred to a slide box and stored in a freezer at −80 °C until further use.

Slides with sections were lyophilized to remove ice and then warmed to room temperature. Epithelial cells of secretory-phase resin ducts from needles were captured using the PALM MicroBeam Axiovert 200 system (Carl Zeiss AG, Oberkochen, Germany) with ×40 magnification and a laser intensity setting between 65 and 75 (focus changed depending on tissue thickness). The laser was directed to cut through neighboring sheath cells to ensure the fidelity of epithelial cells. Mesophyll cells, located further away from resin ducts, were collected in the same way. Microdissected samples were collected in the caps of RNase-free 0.6 ml microcentrifuge tubes with 20 µl of RLT Buffer (RNeasy kit; Qiagen, Hilden Germany). Samples were stored at –80 °C until sufficient cells (2000–2500 cells per sample) had been collected for RNA extraction.

RNA extraction and DNase I treatment were performed using the RNAqueous Total RNA Isolation Kit (Ambion, Thermo Fisher Scientific, Waltham, MA, USA) with minor modifications and based on suggestions by Abbott *et al.* (2010). RNA was precipitated by adding 0.1 volume of 3 M sodium acetate (pH 5.2) and 2.5 volumes of chilled (−20 °C) ethanol to each sample and maintaining the sample at –20 °C for 48 h. Samples were then centrifuged at 15000 *g* for 30 min at 4 °C and the supernatant was discarded. Pellets were washed with 100 µl of 70% aqueous ethanol and centrifuged again at 15000 *g* for 30 min at 4 °C. Microfuge tubes were inverted and pellets were allowed to dry at 23 °C for 10 min before the caps were closed and the samples were packed with dry ice to keep the pellets cold during transit. The Whole-Transcriptome Ovation Pico RNA Amplification System version 1.0 (NuGEN Technologies, San Carlos, CA, USA) was used for cDNA synthesis and linear amplification according to the manufacturer’s instructions. Quality control tests were performed using a Bioanalyzer instrument (Agilent Technologies) according to the manufacturer’s instructions. Single end reads of 50 bp were generated from epithelial and mesophyll cell transcriptome libraries on a HighSeq 2500 instrument (Illumina, San Diego, CA, USA). Contigs were assembled using Trinity (version 2.2.0) (Haas *et al.*, 2013). From six different RNA samples we generated a total of 293 × 10^6^ single-end reads (50 bp long; 147 × 10^6^ from epithelial cells; 145 × 10^6^ from mesophyll cells). Reads were pooled and adapter sequences trimmed using Trimmomatic (Lohse *et al.*, 2012). The trimmed reads were then used to generate a *de novo* transcriptome assembly (combining data from all samples) using Trinotate (version 3.0.0) (https://trinotate.github.io. Last accessed on October 01, 2018.; Haas *et al.*, 2013). The consensus assembly contained 178982 sequences with a mean length of 646 bp. Expression levels were calculated for each individual sample using RSEM (version 1.2.22) (Li and Dewey, 2011) and Bowtie (version 1.0.0) (Langmead, 2010). Annotations [including Gene Ontology (GO) categorization] were generated using Trinotate within the Trinity pipeline (Haas, *et al.*, 2013). Additional assembly statistics are provided in Supplementary Protocol S1.

### Genome-scale modeling of cell-type-specific metabolism

The assumptions and computational approaches employed to generate the Pintae_Epi and Pintae_Meso models are described in Supplementary Protocol S1. One important measurement for these efforts was the determination of the volume of resin produced by epithelial cells. The oleoresin concentration had been determined for bulk needle tissue. For the Pintae_Epi model, it was important to calculate the production of oleoresin by individual resin ducts and their epithelial cells. We therefore determined the volume fraction of needles occupied by resin ducts. Fascicles (bundles with a sheath typically holding three needles) were removed from the main trunk and all branches according to a pattern illustrated in Supplementary Fig. S1. Using a razor blade, cross-sections were obtained by hand in the center of needles. Sections were mounted on glass slides, a drop of mineral oil was added, and sections were viewed under a light microscope (DM LM; Leica Microsystems, Wetzlar, Germany) with camera attachment (EOS Rebel XS; Canon, Tokyo, Japan). Sections were photographed with appropriate rulers for scaling. The area of the cross-section was determined using the ‘Freehand’ tool of ImageJ v.1.45s (https://imagej.nih.gov/ij/index.html. Last accessed on October 01, 2018.) (Supplementary Protocol S1). To convert the needle cross-sectional area into a volume, the tapering of needles at the distal end was taken into account (Supplementary Protocol S1). The volume of the proximal two-thirds was calculated by multiplying the cross-sectional area by the length of the leaf up to that point (volume = area × length). The volume of the remaining third was calculated using the formula for elliptical structures (volume = ⅔ × area × length). The area of each resin duct was determined using the ‘Oval’ tool in ImageJ. The resin duct volume was calculated by treating these structures as a cylinder that extends throughout the length of a needle (volume = area × length) (Supplementary Protocol S1).

## Results

### Epithelial cell transcriptome is enriched in genes of oleoresin biosynthesis

**Fig. 2.**
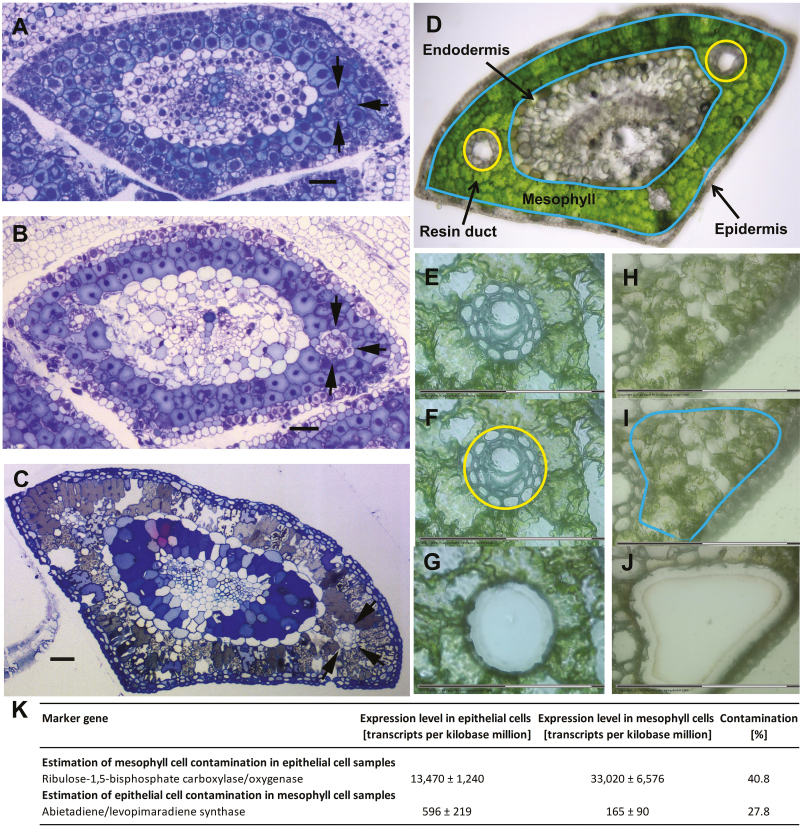
Collection of single cell types for comparative transcriptome analyses. Transverse sections of loblolly pine needles representing (A) a young portion near the intercalary meristem, (B) an older portion of with lateral resin ducts at early secretory phase, and (C) a mature leaf with post-secretory phase resin ducts. Arrows indicate one of the two lateral initiating resin ducts. Bars=100 µm. (D) Epithelial cells surrounding resin ducts were collected by laser-capture microdissection (LCM) at the secretory stage (D) (circles). Mesophyll cells, situated in the area between the epidermis and endodermis (delineated by outlines but excluding the resin ducts), were collected in the same way (D). (E–G) LCM collection of epithelial cells at higher resolution, showing (E) tissue before cell collection, (F) tracing by laser (circle), and (G) tissue after cell capture (G). (H–J) The equivalent process for mesophyll cells is shown: (H) tissue before cell collection, (I) tracing by laser (outline), and (J) tissue after cell capture. (K) Expression levels for the small subunit of Rubisco and abietadiene/levopimaradiene synthase were used to estimate contamination of epithelial cell samples by mesophyll cells and vice versa.

Before describing our sample collection, we will briefly discuss resin duct development in needles. There are always two lateral ducts, on opposite sides of the needle and one cell layer removed from the endodermis, which surround the transfusion tissue of the central vascular cylinder (Fig. 2A). Occasionally, additional ducts form at the adaxial and abaxial sides of the central cylinder. We collected only the lateral ducts for our study (Fig. 2B). During development, needles continue to grow from the activity of a basal intercalary meristem near the base (Werker and Fahn, 1969), so that mature tissues can often be found in the mid- and apical regions along the needle, while newly differentiated tissue occurs near the base. Newly differentiated regions on elongated ducts form in direct contact with more mature regions. Developing resin ducts are therefore lined by regular rows of differentiating cells of different developmental ages (Fahn, 1988). A developing resin duct thus presents a linear progression of epithelial cell development, including pre-secretory, secretory, and post-secretory stages. For the current study, epithelial cells were collected at the secretory stage (corresponding to Fig. 2C), while attempting to avoid the collection of cells at the post-secretory stage (Fig. 2D).

Epithelial cells of resin ducts at the secretory stage were collected from needle cryosections by laser-capture microdissection (LCM) (Fig. 2E–G). As a reference, adjacent mesophyll cells, which we hypothesized would be less active in oleoresin biosynthesis, were obtained in the same way (Fig. 2H–J). RNA was extracted from pooled cells (~2500 cells per pool) representing each single cell type (epithelial and mesophyll cells collected separately) of needles of three different saplings. RNA was then amplified linearly and subjected to transcriptome sequencing by RNA-Seq. Sequence fragments were assembled into contigs and functionally annotated, and the abundance of transcripts represented by these contigs was calculated (Supplementary Table S1). We then estimated potential cross-contamination of the two sample sets. To assess the possible contamination of our epithelial cell transcriptome data by transcripts that might represent mesophyll cell contamination, we compared the expression levels for the small subunit of ribulose 1,5-bisphosphate carboxylase/oxygenase (Rubisco); the rationale for this was that epithelial cells are a non-green cell type (Charon *et al.*, 1987; Cheniclet and Cardé, 1985) and Rubisco is generally the most abundant protein in photosynthetic cell types such as mesophyll cells. There was a significant abundance of Rubisco small subunit transcripts in epithelial cells [13470 transcripts per kilobase million (TPM); as a reference, the abundance in mesophyll cells was 33020 TPM], which, if it were assumed that these resulted entirely from contamination, would indicate a 40.8% contribution of mesophyll cells to the epithelial cell transcriptome (Fig. 2K). To evaluate the reverse (epithelial cell contamination of mesophyll transcriptome data sets), we compared the transcript abundance values for abietadiene/levopimaradiene synthase (LAS); the rationale for this choice was that the corresponding enzyme has been demonstrated to localize exclusively to epithelial cells in Norway spruce (Keeling and Bohlmann, 2006). The gene expression values for LAS (165 and 596 TPM in mesophyll and epithelial cells, respectively) would represent a 27.8% contribution of epithelial cells to the mesophyll cell transcriptome (Fig. 2K).

Statistical processing of the RNA-Seq data sets identified 212 overexpressed and 275 repressed transcripts in epithelial cells (compared with mesophyll cell controls and not considering possible contamination). A GO enrichment analysis indicated that the terms ‘epithelial cell proliferation’ (GO:0050678), ‘terpene synthase activity’ (GO:0010333), ‘monoterpene biosynthetic process’ (GO:0043693), and ‘geranyl diphosphate metabolic process’ (GO:0033383) were uniquely present among genes with significantly higher expression levels in epithelial cells, when compared with mesophyll cells (Supplementary Table S2), indicating a possible specialization of the secretory cell type. We then assessed whether this notion was supported by analyses of expression patterns of genes known to be involved in terpenoid biosynthesis.

### Most MEP pathway genes are transcriptionally up-regulated in epithelial cells

**Fig. 3.**
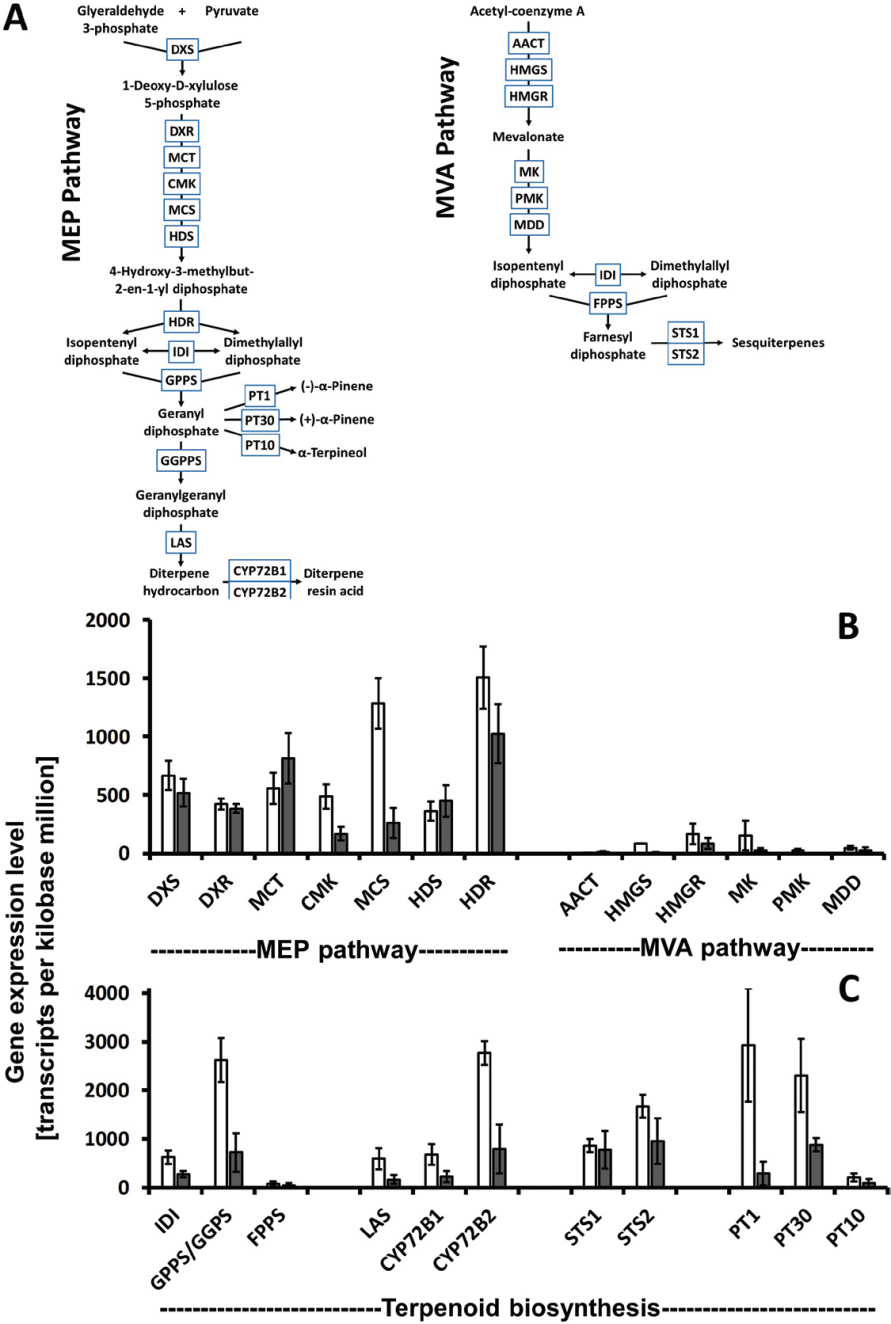
Genes involved in oleoresin biosynthesis are enriched in epithelial cells, when compared to mesophyll cell controls. (A) Overview of relevant biosynthetic pathways. Gene expression patterns of (B) precursor supply pathways and (C) terpenoid-specific pathways are shown separately. Bars represent the SE of three replicate samples. AACT, acetoacetyl-CoA thiolase; CMK, 4-(cytidine 5ʹ-diphospho)-2-C-methyl-D-erythritol kinase; CYP, cytochrome P450 oxygenase; DXR, 1-deoxy-D-xylulose 5-phosphate reductoisomerase; DXS, 1-deoxy-D-xylulose 5-phosphate synthase; FPPS, farnesyl diphosphate synthase; GGPPS, geranylgeranyl diphosphate synthase; GPPS, geranyl diphosphate synthase; HDR, 1-hydroxy-2-methyl-2-butenyl 4-phosphate reductase; HDS, 1-hydroxy-2-methyl-2-butenyl 4-phosphate synthase; HMGR, 3-hydroxy-3-methylglutaryl-CoA reductase; HMGS, 3-hydroxy-3-methylglutaryl-CoA synthase; IDI, isopentenyl diphosphate isomerase; LAS, abietadiene/ levopimaradiene synthase; MCS, 2-C-methyl-D-erythritol 2,4-cyclodiphosphate synthase; MCT, 2-C-methyl-D-erythritol 4-phosphate cytidylyltransferase; MDD, mevalonate 5-diphosphate decarboxylase; MEP, 2-C-methyl-D-erythritol 4-phosphate; MK, mevalonate kinase; MVA, mevalonate; PMK, phosphomevalonate kinase; PT1, (–)-α-pinene synthase; PT10, α-terpinene synthase; PT30, (+)-α-pinene synthase; STS, sesquiterpene synthase.

Genes of the 2-C-methyl-D-erythritol 4-phosphate (MEP) pathway, which primarily provides precursors for monoterpene (C10) and diterpene (C20) biosynthesis (major end products accumulating in resin ducts) (Fig. 3A), were expressed at fairly high levels in epithelial cells (360–1505 TPM). In mesophyll cells, where precursors for the biosynthesis of chlorophylls and carotenoids are derived from the MEP pathway (Banerjee and Sharkey, 2014), these genes were expressed at comparable levels (167–1024 TPM) (Fig. 3B). The abundance of transcripts related to 4-diphosphocytidyl-2-C-methyl-D-erythritol kinase and 2-C-methyl-D-erythritol 2,4-cyclodiphosphate synthase was significantly higher in epithelial cells (486 and 1287 TPM, respectively) compared with mesophyll cells (167 and 260 TPM, respectively). The expression levels of the other MEP pathway genes were generally higher in epithelial cells than in mesophyll cells, but this difference was not statistically significant (*P*-value in two-tiered *t*-test not <0.05). The only exception was the gene coding for 2-C-methyl-D-erythritol 4-phosphate cytidylyltransferase, which was expressed at higher levels in mesophyll cells (814 TPM) compared with epithelial cells (556 TPM).

Genes of the mevalonate (MVA) pathway, which is generally responsible for generating precursors of sesquiterpenes (C15) and sterols (C30) (Fig. 3A), were expressed at low levels in both sample types (0–163 TPM) (Fig. 3B). Statistically significant differences were determined for 3-hydroxy-3-methylgluytaryl coenzyme A synthase (83 and 5 TPM in epithelial and mesophyll cells, respectively) and mevalonate kinase (149 and 20 TPM in epithelial and mesophyll cells, respectively) (Fig. 3B). Genes encoding C10 and C20 prenyltransferases [geranyl diphosphate synthase (GPPS small subunit) and geranylgeranyl diphosphate synthase (GGPPS)] were highly abundant in epithelial cells (total of 2620 TPM) and moderately abundant in mesophyll cells (total of 718 TPM), while transcripts corresponding to C15 farnesyl diphosphate synthase (FPPS) were of low abundance in both sample types (85 and 53 TPM in epithelial and mesophyll cells, respectively) (Fig. 3C).

Genes directly involved in diterpene resin acid biosynthesis [coding for LAS (Ro and Bohlmann, 2006), CYP720B1 (a multifunctional oxygenase that generates resin acids; Ro *et al.*, 2005) and a putative ortholog of CYP720B2 (also a resin acid-generating oxygenase; Geisler *et al.*, 2016)] were expressed at fairly high levels in epithelial cells (596, 675 and 2769 TPM, respectively), with much lower abundance in mesophyll cells (165, 225 and 793 TPM, respectively) (Fig. 3C). The gene encoding a putative sesquiterpene synthase (STS1, similar to *Picea abies* longifolene synthase) was expressed at comparable levels in epithelial cells (861 TPM) and mesophyll cells (776 TPM). A second gene coding for a putative sesquiterpene synthase (STS2, similar to *Abies grandis* δ-selinene synthase) was expressed at higher levels in epithelial cells (1671 TPM) compared with mesophyll cells (947 TPM) (Fig. 3C). Transcripts directly related to monoterpene formation, in particular (–)-α-pinene synthase (PT1) and (+)-α-pinene synthase (PT30), whose encoded enzymes generate the major monoterpenes of loblolly pine oleoresin, were highly abundant in epithelial cells (2933 and 2301 TPM, respectively) and were of much lower abundance in mesophyll cells (242 and 130 TPM, respectively) (Fig. 3C).

### Assessing differences in flux distribution between epithelial and mesophyll cells by genome-scale modeling

**Fig. 4.**
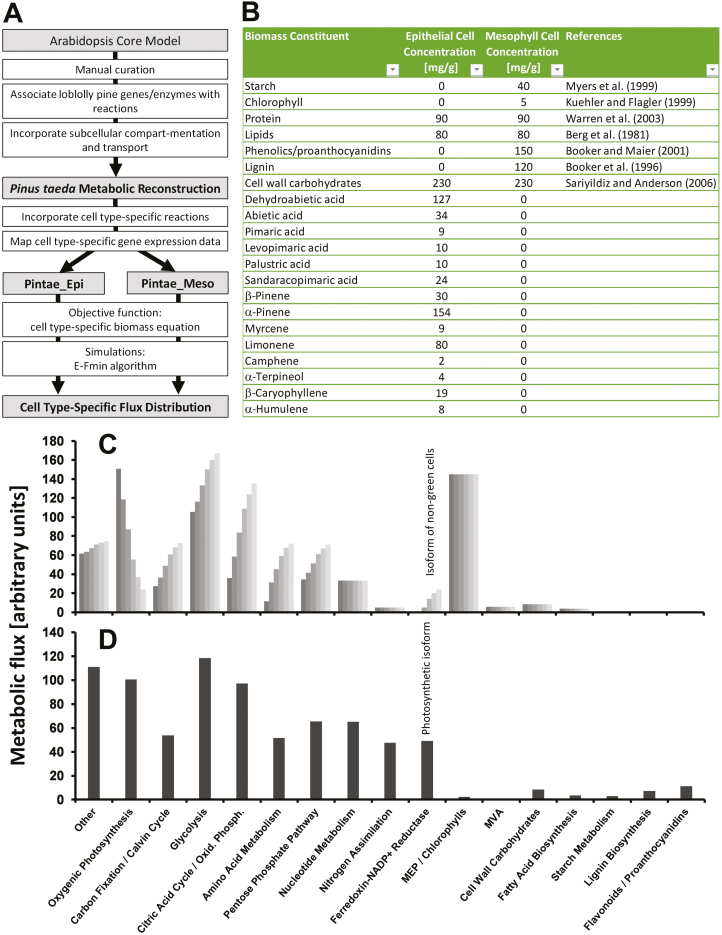
Genome-scale modeling captures the metabolic specialization of loblolly pine epithelial cells. (A) Development of cell-type-specific models for epithelial cells (Pintae_Epi) and mesophyll cells (Pintae_Meso) of loblolly pine needles. (B) Biomass outputs used in objective functions in the Pintae_Epi and Pintae_Meso models. The oleoresin measurements (left data column) were conducted as part of this study. The data sets are included in Supplementary Table S3. Simulations were performed by using an objective function that considers the different biomass outputs of epithelial and mesophyll cells. For details see Supplementary Protocol S1. (C, D) Predicted flux distribution through metabolic pathways in (C) epithelial cells at different levels (left to right: 100, 75, 50, 25, 10, and 0%) of photosynthetic activity and (D) mesophyll cells (assuming fully active photosynthesis).

To further assess metabolism in loblolly pine needles, we performed a genome-scale metabolic reconstruction, which we describe briefly here (for a detailed description see Supplementary Protocol S1). This model was developed based on a previously published, well-curated model for photosynthetic tissues in *Arabidopsis thaliana* L. (Arabidopsis Core Model) (Arnold and Nikoloski, 2014). The EC numbers for the enzymes catalyzing the reactions represented in our reconstruction were transferred from the Arabidopsis Core Model. Searches against online databases pertaining to plant metabolism [MetaCyc version 19.1 (Caspi *et al.*, 2016) and AraCyc version 21.5 (Lamesch *et al.*, 2012)], augmented by traditional literature searches, were then conducted to assess the current knowledge regarding metabolic reactions in loblolly pine that differ from those known to occur in Arabidopsis or are uniquely present in conifers (e.g. oleoresin biosynthesis) (Fig. 4A). Each reaction of the metabolic reconstruction was associated with a compartment (cytosol, plastid, mitochondrion, or peroxisome) based on predictions, using several web-based tools (Emanuelsson *et al.*, 2007), for subcellular localization of the relevant enzyme (Fig. 4A). Additional steps involved removing thermodynamically infeasible loops (to ensure a stoichiometrically balanced model) and eliminating reactions that cannot carry flux (orphans not connected to the remainder of the metabolic network) (see Supplementary Protocol S1 for details).

The contigs of the loblolly pine epithelial cell transcriptome assembly were compared, using the Blastx algorithm, to the UniProt and TAIR sequences represented in the MetaCyc and AraCyc databases, respectively. Loblolly pine transcripts were associated with reactions from the Arabidopsis Core Model, AraCyc, or MetaCyc (in this order of priority) on the basis of global identity, and this information was transferred to our metabolic reconstruction. This process also allowed us to fetch EC numbers and the GO annotation for each gene/enzyme associated with a particular reaction. Reactions in the metabolic reconstruction with no associated transcripts were removed. These additional steps generated the loblolly pine epithelial cell metabolic model (total of 694 reactions; Supplementary Table S3), which we termed Pintae_Epi (Fig. 4A). Experimentally determined concentrations of oleoresin constituents and estimated concentrations of other end products of metabolism were integrated into Pintae_Epi as part of an objective function (treating the generation of biomass constituents as the ‘objective’ of the metabolic network) (Fig. 4B). Detailed considerations for developing objective functions for secretory cell types are provided in Johnson *et al.* (2017). The incorporation of metabolically synthesized amino acids into total protein was calculated based on the amino acid composition of glutathione *S*-transferase, the enzyme encoded by the most highly expressed non-photosynthetic gene of this cell type. Using the same general approach, a separate model for mesophyll cells, termed Pintae_Meso, was also generated (total of 722 reactions; Fig. 4A). The incorporation of metabolically synthesized amino acids into total protein was calculated based on the amino acid composition of Rubisco, the enzyme encoded by the most highly expressed gene in this cell type. The E-Fmin algorithm (Song *et al.*, 2014) was then employed to obtain predictions for flux minimization, as a function of weighted gene expression values, through the metabolic networks of the Pintae_Epi and Pintae_Meso models, while satisfying each objective function.

Simulations with Pintae_Epi were performed based on the following assumptions: (i) the import of sucrose as a carbon source was enabled to mirror the heterotrophic metabolism previously demonstrated to occur in epithelial cells (Cheniclet and Cardé, 1985; Charon *et al.*, 1987; Langenfeld-Heyser, 1987) and (ii) photosynthesis was allowed to occur to account for the fact that our epithelial cell transcriptome data indicated the presence of transcripts related to this process. However, since previous studies indicated that epithelial cells contain non-photosynthetic leucoplasts (Cheniclet and Cardé, 1985; Charon *et al.*, 1987), we simulated various levels of photosynthetic activity (at 100, 75, 50, 25, and 0% of the theoretical photosynthetic efficiency) to assess the implications of mixed sources of carbon (from primarily photosynthetic CO_2_ fixation to metabolism that is entirely dependent on an imported transport oligosaccharide). As expected, the flux through oxygenic photosynthesis decreased gradually with decreased conversion of energy from photons (with only the plastidial ATPase carrying significant flux under non-photosynthetic conditions) (Fig. 4C). In contrast, as the contribution of photosynthesis decreased, flux through the pentose phosphate pathway, glycolysis, and amino acid metabolism were predicted to increase. The most notable increase in flux with decreasing levels of photosynthesis was predicted to occur through the citric acid cycle and oxidative phosphorylation (Fig. 4C). Another significant shift was predicted to affect ferredoxin NADP^+^ reductase (FNR). The FNR isoform of photosynthetic cells primarily transfers electrons from photosystem I to NADPH, which is then used as a reducing equivalent in the reactions of the Calvin–Benson cycle; in contrast, the FNR isoform of non-photosynthetic cells operates in reverse, independently of photosystem I, to provide reduced ferredoxin (Aliverti *et al.*, 2008). Our model predicted a gradual transition from primarily photosynthetic to non-photosynthetic FNR with decreasing photosynthetic activity (Fig. 4C).

Simulations were also performed to assess flux distribution through the reactions of the Pintae_Meso model. The main assumption in this case was that mesophyll cells rely primarily on oxygenic photosynthesis, coupled with carbon fixation through the Calvin–Benson cycle, to generate precursors of central carbon metabolism (Öquist and Huner, 2003), and an uptake of a transported oligosaccharide was therefore not considered. The most obvious differences in the flux distribution, compared with the Pintae_Epi simulations, were significantly higher flux through the reactions of oxygenic photosynthesis, dramatically reduced flux through the MEP pathway, and output fluxes to additional end products (e.g., chlorophylls, starch, lignins, and flavonoids/proanthocyanidins) (Fig. 4D).

**Fig. 5.**
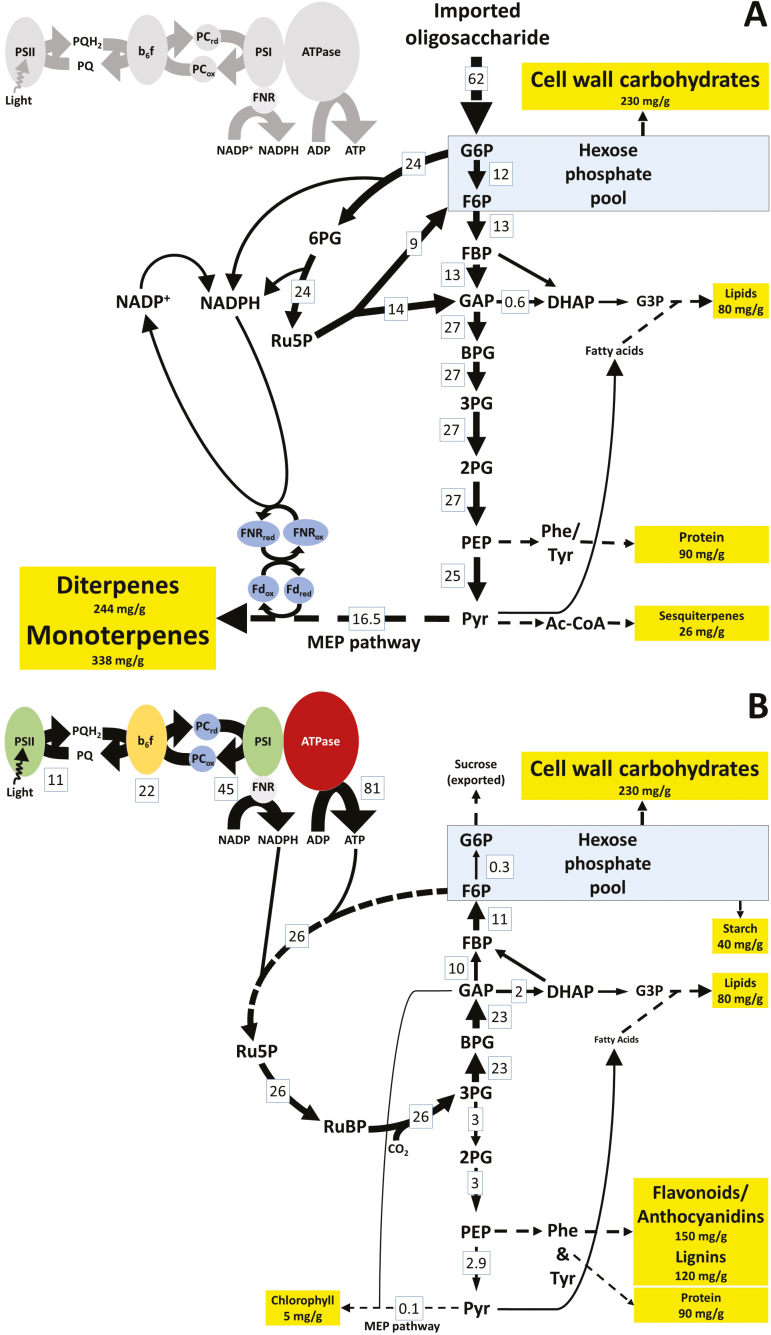
Metabolism of loblolly pine needle epithelial cells shares commonalities with the metabolism of other secretory cell types. Predicted flux distribution through central carbon metabolism in (A) epithelial and (B) mesophyll cells. The thickness of the arrows indicating reactions is proportional to flux (relative fluxes are also indicated numerically). ADP, adenosine diphosphate; ATP, adenosine triphosphate; BPG, 1,3-bisphosphoglycerate; DHAP, dihydroxyacetone phosphate; FBP, fructose 1,6-bisphosphate; FNR, ferredoxin NADP^+^ reductase; G3P, glycerol 3-phosphate; GAP, glyceraldehyde 3-phosphate; Glc, glucose; G6P, glucose 6-phosphate; 6PG, 6-phosphogluconolactone; Gn6P, gluconate 6-phosphate; MEP, 2-C-methyl-D-erythritol 4-phosphate; MVA, mevalonate; NADP, nicotinamide adenine dinucleotide phosphate (oxidized); NADPH, nicotinamide adenine dinucleotide phosphate (reduced); PC, plastocyanin; PEP, phosphoenolpyruvate; Phe, phenylalanine; 2PG, 2-phosphoglycerate; 3PG, 3-phosphoglycerate; PSI, photosystem I; PSII, photosystem II; Pyr, pyruvate; PQ, plastoquinone (oxidized); PQH2, plastoquinone (reduced); Ru5P, ribulose 5-phosphate; RuBP, ribulose 1,5-bisphosphate; Tyr, tyrosine.

Our simulations predicted that in epithelial cells, where an imported carbohydrate is the primary carbon source, a fairly direct conversion through glycolysis and the MEP pathway will generate monoterpenes and diterpenoids as the primary constituents of oleoresins (Fig. 5A). The predicted flux distribution was very different for mesophyll cells, in which the primary carbon source is carbon dioxide, which is assimilated at high flux via the reactions of the Calvin–Benson cycle into central carbon metabolism. In comparison to epithelial cells, in mesophyll cells a larger number of metabolic end products is generated at lower concentrations, which might explain a more even distribution of flux (Fig. 5B).

## Discussion

### Transcriptome data reflect the metabolic specialization of epithelial cells

Abbott *et al.* (2010) used LCM to obtain samples from white spruce (*Picea glauca* L.) stems that enabled gene expression, enzyme activity, and metabolite analyses at tissue-level resolution. Based on the initial demonstration that LAS is localized exclusively to the epithelial cells of resin ducts in Norway spruce (*Picea abies* L.) (Zulak *et al.*, 2010), we collected loblolly pine epithelial cells and mesophyll cells separately by LCM and performed RNA-Seq analyses. Most genes known to be involved in oleoresin biosynthesis (monoterpene synthases, diterpene synthases, and monoterpene oxygenases) were highly enriched in the three replicate transcriptomes of epithelial cells compared with those obtained from mesophyll cell). The expression levels of these genes in mesophyll cells was generally at a level that could be explained by cross-contamination from neighboring epithelial cells (this contamination was estimated at ~28%). The exceptions were two genes coding for putative sesquiterpene synthases, for which transcript abundance was higher in mesophyll cells than would be expected from epithelial cell contamination (Fig. 3). A possible explanation for this observation would be that sesquiterpene biosynthesis occurs in mesophyll cells. It should also be noted that the concentration of sesquiterpenes in loblolly pine oleoresins is very low (constituting less than 5% of total terpenoids) (Tables 1 and 2), and one might therefore expect the expression levels of sesquiterpene synthases with direct relevance for oleoresin biosynthesis to be low and not notably different from those in mesophyll cells. Transcripts related to terpenoid precursor supply via the MEP pathway were present at medium to high abundance in both epithelial and mesophyll cells (Fig. 3). Oleoresin monoterpenes and diterpenes are the main end products in epithelial cells, whereas chlorophylls and carotenoids are the primary products derived from intermediates of the MEP pathway in mesophyll cells. Given the significant differences in predicted flux toward these terpenoid end products in the two cell types (16 and 0.1 in epithelial and mesophyll cells, respectively), the comparatively high expression levels of the MEP pathway genes in mesophyll cells cannot be explained entirely by cross-contamination. This observation can be interpreted as indirect evidence that flux through the MEP pathway might be regulated to a significant extent by post-transcriptional processes, which has been demonstrated to occur in several experimental systems (Banerjee and Sharkey, 2014).

**Table 1.** Volatile constituents of oleoresins obtained from loblolly pine trees

Metabolite	GC-FID	GC-MS			GC-FID		Composition of distillates					
	(DB-WAX)	(DB-5MS)			(CYCLODEX-B)		(% of total volatiles)					
	Rt	Rt	MS library score (%)		Rt	Rt	Needles (*n*=40)		Bark (*n*=24)		Xylem (*n*=29)	
			Adams	Auth Std	(–)-Enant	(+)-Enant	(–)-Enant	(+)-Enant	(–)-Enant	(+)-Enant	(–)-Enant	(+)-Enant
α-Pinene	5.13	6.87	97	98	17.17	17.70	9.04 ± 1.13	48.86 ± 0.31	5.20 ± 0.70	20.33 ± 7.61	7.95 ± 1.10	40.89 ± 8.12
Camphene	5.73	7.23	95	97	19.17	19.63	0.35 ± 0.07	0.48 ± 0.03	0.26 ± 0.11	0.23 ± 0.07	0.43 ± 0.16	0.52 ± 0.13
β-Pinene	6.40	8.00	96	98	20.79	20.63	25.81 ± 3.50	0.60 ± 0.09	14.85 ± 4.30	0.28 ± 0.09	32.16 ± 6.17	0.70 ± 0.16
Sabinene	6.54	7.92	67	96	18.50	18.70	0.37 ± 0.04	<0.1	0.24 ± 1.00	0.13 ± 0.07	0.36 ± 0.11	<0.1
Limonene	8.23	9.53	97	98	21.70	21.90	1.68 ± 0.17	0.35 ± 0.04	12.72 ± 6.17	0.55 ± 0.12	5.03 ± 1.91	0.49 ± 0.15
Myrcene	7.16	8.22	95	97	18.07±		2.43 ± 0.11		9.07 ± 4.81		2.01 ± 0.29	
β-Phellandrene	8.49	9.62	95	97	22.71±		1.11 ± 0.21		5.19 ± 3.20		1.17 ± 0.34	
β-Caryophyllene	22.96	25.65	99	99	46.70±		3.36 ± 0.88		3.29 ± 0.28		0.30 ± 0.11	
α-Humulene	26.99	27.09	98	98	48.61±		0.70 ± 0.14		0.71 ± 0.16		<0.1	
α-Terpineol	28.74	16.02	64	96	40.92±		0.62 ± 0.18		0.62 ± 0.18		0.34 ± 0.10	
Germacrene D	29.50	28.12	99	99	49.88±		2.68 ± 1.20		5.66 ± 1.37		0.45 ± 0.13	
							**Concentration of volatiles (mg g** ^–1^)					
							**Needles (*n*=40**)		**Bark (*n*=24**)		**Xylem (*n*=29**)	
Monoterpenes							5.76 ± 1.68		2.99 ± 1.31		3.68 ± 2.73	
Sesquiterpenes							0.35 ± 0.11		0.18 ± 0.09		0.02 ± 0.02	
Total volatiles							6.11 ± 1.75		3.17 ± 1.36		3.69 ± 2.75	

Auth Std, authentic standard; Enant, enantiomer; GC-FID, gas chromatography with flame ionization detection; Rt, retention time.

**Table 2.** Semi-volatile constituents of oleoresins obtained from loblolly pine trees

Metabolite	GC-MS (DB-5MS)			Composition		
	Rt	MS library score (%)		(% of semi-volatiles)		
		Adams	Auth Std	Needles (*n*=40)	Bark (*n*=24)	Xylem (*n*=29)
**Olefins**						
Isopimaradiene	43.23	92	97	n.d.	n.d.	n.d.
Pimaradiene	44.67	89	96	n.d.	n.d.	n.d.
Sandaracopimaradiene	45.35	93	98	0.23 ± 0.08	n.d.	n.d.
Levopimaradiene	47.19	89	99	n.d.	n.d.	n.d.
Dehydroabietadiene	48.16	88	98	n.d.	n.d.	n.d.
Abietadiene	49.20	94	97	n.d.	n.d.	n.d.
Neoabietadiene	51.25	94	98	0.33 ± 0.11	n.d.	n.d.
**Alcohols**						
Sandaracopimaradienol	54.61	82	97	n.d.	n.d.	0.15 ± 0.08
Isopimaradienol	55.76	73	n.a.	n.d.	n.d.	n.d.
Palustradienol	56.39	69	n.a.	n.d.	n.d.	n.d.
Dehydroabietadienol	57.43	78	98	0.83 ± 0.24	1.01 ± 0.41	n.d.
Abietadienol	58.36	79	97	n.d.	7.32 ± 2.33	n.d.
Neoabietadienol	60.16	67	n.a.	n.d.	n.d.	n.d.
**Cyclic ethers**						
Manoyl oxide	46.42	67	92	n.d.	0.99 ± 0.32	n.d.
epi-Manoyl oxide	47.09	59	94	n.d.	3.67 ± 0.65	n.d.
**Aldehydes**						
Sandaracopimaradienal	52.09	78	n.a.	0.56 ± 0.16	n.d.	0.54 ± 0.18
Dehydroabietadienal	54.73	72	n.a.	0.27 ± 0.12	0.20 ± 0.09	0.21 ± 0.09
Abietadienal	55.86	66	n.a.	0.59 ± 0.12	0.68 ± 0.12	n.d.
**Resin acids (as methyl esters**)						
Pimaric acid	53.55	93	98	7.76 ± 3.59	12.74 ± 5.64	14.70 ± 3.83
Sandaracopimaric acid	54.05	93	98	12.31 ± 3.19	6.71 ± 1.36	4.00 ± 0.75
Isopimaric acid	55.31	92	97	4.44 ± 2.46	6.36 ± 2.74	11.99 ± 5.79
Levopimaric/palustric acid	55.50	95	99	3.35 ± 2.67	5.65 ± 5.73	16.83 ± 8.27
Dehydroabietic acid	56.49	94	99	33.45 ± 7.82	31.07 ± 8.16	29.54 ± 7.57
Abietic acid	57.83	94	97	26.56 ± 6.00	30.81 ± 6.36	17.57 ± 6.39
Neoabietic acid	59.13	93	96	9.40 ± 7.20	5.29 ± 5.13	4.48 ± 1.94
				**Concentration of semi-volatiles (mg g** ^**–1**^)		
				**Needles (*n*=40**)	**Bark (*n*=24**)	**Xylem (*n*=29**)
Resin acids				3.81 ± 1.13	5.65 ± 2.41	6.66 ± 3.68
Other diterpenoids				0.11 ± 0.01	0.40 ± 0.17	0.06 ± 0.01
Total diterpenoids				3.92 ± 1.09	6.04 ± 2.33	6.72 ± 3.67

Auth Std, authentic standard; GC-MS, gas chromatography–mass spectrometry; n.a., no authentic standard available (quantities estimated); n.d., not detectable; Rt, retention time.

We encountered additional unexpected findings during the evaluation of our transcriptome data sets. For example, transcripts related to photosynthesis and carbon fixation were clearly present in our epithelial cell transcriptome, although this cell type had previously been demonstrated to lack functional photosystems (Cheniclet and Cardé, 1985; Charon *et al.*, 1987). This result is consistent with recent data from *Picea glauca*, which also indicated the presence of transcripts related to photosynthesis in various bark cell types, including epithelial cells (Celedon *et al.*, 2017). This observation might be a reflection of the conservative approach we (and others) have taken to ensure that the cell type of interest remained intact during LCM, which for the case of epithelial cells entailed cutting through sheath cells and possibly also some photosynthetic mesophyll cells. In other words, the observed expression levels of genes related to photosynthesis and carbon fixation might partly be an artefact of the cell collection method. However, we found that our GO analysis also indicated that genes involved in chlorophyll breakdown (GO:0015996, GO:0046149, GO:0006787, and GO:0033015) were significantly enriched in the epithelial cell transcriptome, while those related to chlorophyll biosynthesis (GO:0034256, GO:0033354, and GO:0090415) were enriched in the mesophyll cell data set (Supplementary Table S2). It is therefore conceivable that components of the photosynthetic machinery are synthesized in epithelial cells but are then broken down or not fully assembled. Further research, beyond the scope of this study, will be required to evaluate these intriguing possibilities.

### Predicted flux distribution is strikingly similar across secretory cell types

We developed a reconstruction of metabolism in epithelial cells of loblolly pine needles and integrated our single cell transcriptome data to develop the Pintae_Epi model. Fluxes were then predicted by calculating the minimum net flux through the network, while satisfying the biomass equation (which contains measured and estimated metabolite concentrations as outputs). When modeling conditions were adjusted so that imported sucrose served as the primary carbon source (with varying degrees of photosynthetic activity), flux increased significantly through glycolysis, which enables efficient conversion into precursors for the biosynthesis of terpenoids, the major constituents of loblolly pine oleoresins (Fig. 5). Considerable flux is also predicted to be carried by the oxidative pentose phosphate pathway, which generates essential reducing equivalents when photosynthesis does not. The main pathway to carry flux toward end products in epithelial cells is the MEP pathway, the products of which are accumulated to significantly higher concentrations than all other end products. In contrast, mesophyll cells are predicted to utilize primarily photosynthesis coupled with the Calvin–Benson cycle to generate precursors of central carbon metabolism (Fig. 5). These are then incorporated into a variety of metabolic end products in mesophyll cells, but all at much lower concentrations than oleoresins in epithelial cells. It would thus appear that the specialization of epithelial cells for high oleoresin biosynthesis is made possible by a ‘rewiring’ of the metabolic network that favors the most direct route to provide terpenoid precursors. This conclusion is consistent with recent data that demonstrated that terpenoid biosynthesis in photosynthetic glandular trichomes of tomato depends primarily on an imported carbon source (Balcke *et al.*, 2017).

A particularly high flux was predicted to occur through the MEP pathway of epithelial cells, where two reactions—those catalyzed by the iron-sulfur cluster enzymes 1-hydroxy-2-methyl-2-(*E*)-butenyl-4-diphosphate synthase and 1-hydroxy-2-methyl-2-(*E*)-butenyl-4-diphosphate reductase—require electron transfer through the ferredoxin/FNR system. We recently demonstrated the presence of a unique pair of ferredoxin and FNR isoforms in secretory cells of peppermint glandular trichomes (Johnson *et al.*, 2017). The insufficient sequencing depth of our transcriptome data and the paucity of literature reporting on conifer ferredoxin and FNR prevented us from assessing whether epithelial cells of loblolly pine resin ducts may contain a similar arrangement of isoforms. We also demonstrated previously that both oxidative phosphorylation and fermentation were highly active to recycle energy equivalents in non-photosynthetic peppermint glandular trichomes (Johnson *et al.*, 2017). Genes involved in both processes were found to be enriched in the loblolly pine epithelial cell transcriptome (compared with mesophyll cells) (Supplementary Table S4). It would therefore seem that there are intriguing commonalities across plant lineages with regard to flux distribution in heterotrophic cell types that evolved the capacity to synthesize terpenoids and excrete them into an extracellular storage cavity. Our data sets have laid the foundation for follow-up studies to identify the genetic basis for the specialization of these cell types in diverse plant lineages. Such studies have the potential to unravel the tools needed to improve the accumulation of oils and resins, which have important roles in ecology and as precursors for commercial products.

## Data deposition

The raw transcriptome sequence data for epithelial cells and mesophyll cells of loblolly pine needles are available at the NCBI Sequence Read Archive, accession number SRP126587 (https://www.ncbi.nlm.nih.gov/sra. Last accessed on October 01, 2018.).

## Supplementary data

Supplementary data are available at *JXB* online.

**Fig. S1.** Schematic of the collection of tissue types for oleoresin analysis and the peeling of bark from stems.

**Protocol S1.** Detailed descriptions of methods and approaches for building the Pintae_Epi and Pintae_Meso genome-scale metabolic models.

**Table S1.** Loblolly pine epithelial and mesophyll cell transcriptome assemblies (with functional annotation).

**Table S2.** Gene Ontology enrichment analysis of transcriptomes obtained with loblolly pine epithelial cells and mesophyll cells.

**Table S3.** Genome-scale models of metabolism in loblolly pine epithelial cells (Pintae_Epi) and mesophyll cells (Pintae_Meso).

**Table S4.** Genes involved in fermentative reactions and the electron transport chain (regeneration of energy equivalents) are enriched in epithelial cells compared with mesophyll cell controls.

## Supplementary Material

Supplementary Table S1

Supplementary Table S2

Supplementary Table S3

Supplementary Table S4

Supplementary Figure S1 and Protocol S1
