# Supplementary material for: Assessment of flux through oleoresin biosynthesis in epithelial cells of loblolly pine resin ducts
Source: J Exp Bot. 2018 Oct 11;70(1):217–30. doi: 10.1093/jxb/ery338 (PMC6305192; doi:10.1093/jxb/ery338)
Supplement: Supplementary Figure S1 and Protocol S1 [file ery338_suppl_figure_s1_protocol_s1.pdf]

Supplementary Fig. S1. Collection of tissue types for oleoresin analysis (A), with orange squares indicating the locations for harvesting xylem and bark tissues, and blue squares indicating the locations for needle harvests. The peeling of bark from stems is illustrated in panel (B).

A **B**

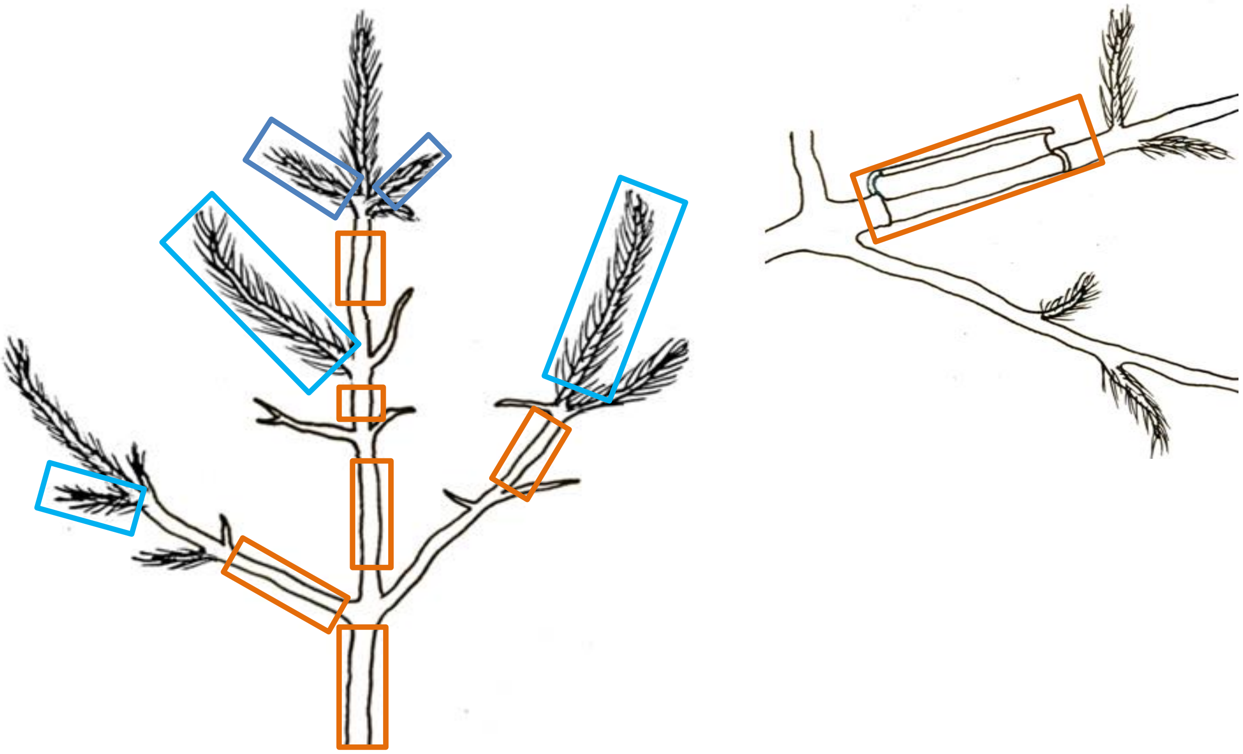

Supplementary Protocol S1

Turner et al. (2018) Assessing Flux Through Oleoresin Biosynthesis in Epithelial Cells of Loblolly Pine Resin Ducts. *Journal of Experimental Botany*.

1. Development of Genome-Scale Models of Secretory-Stage Epithelial Cells of Resin Ducts and Mesophyll Cells

1.1 Metabolic Reconstruction

The Pintae_Epi model was created to computationally predict flux distribution through metabolic pathways of epithelial cells of resin ducts in loblolly pine (*Pinus taeda* L.) needles. As a first step, primary metabolic reactions, including stoichiometry, were adopted from the well-curated *Arabidopsis* Core Model (Arnold and Nikoloski, 2014) (Scheme 1). Searches against several online databases pertaining to plant metabolism (MetaCyc Version 19.1, http://metacyc.org/ (Caspi *et al.*, 2014) and AraCyc Version 15, https://www.plantcyc.org/databases/aracyc/15.0 (Lamesh *et al.*, 2012)), augmented by traditional literature searches, were then conducted to assess the current knowledge regarding metabolic reactions in loblolly pine that differ from those known to occur in *Arabidopsis* (e.g., oleoresin biosynthesis). The E.C. numbers for the enzymes catalyzing the reactions represented in Pintae_Epi were transferred from the Arabidopsis Core Model or assigned manually based on the relevant literature on oleoresin biosynthesis. Each gene (and corresponding enzyme/reaction) was assigned to metabolic processes and pathways based on the Gene Ontology annotation of the most similar gene contained in the *Arabidopsis* genome (https://www.arabidopsis.org/tools/bulk/go/). The same process was employed to develop a metabolic reconstruction for mesophyll cells (Scheme 1).

1.2 Incorporation of Subcellular Compartmentation and Transport

Each of the reactions of the metabolic reconstructions was associated with a subcellular compartment (cytosol, plastid, mitochondrion, or peroxisome) based on the known or predicted localization of the enzyme encoded by the putative *Arabidopsis* ortholog (Hooper *et al.*, 2014) (Scheme 1). The model allows for free exchange of O_2 and water into and out of the cell, and contains a single light "import" reaction that is irreversible. Sucrose is the only nutrient that can freely enter into the cell (but not out; acconting for a proton symport mechanism), while others, such as NO_3^-, PO_4^{3-}, and SO_4^{2-}, are imported at the expense of ATP and water (in stoichiometrically balanced reactions). All amino acids are generated *de novo* from intermediates of central carbon metabolism and nitrogen ultimately coming from imported NO_3^-. Roughly 40% of all reactions are transport reactions, which are responsible for the exchange of metabolites between compartments, while the remaining 60% of reactions are involved in the biosynthesis or degradation of metabolites.

1.3 Refinements

Additional steps involved removing thermodynamically infeasible loops (which ensures a stoichiometrically balanced model) and eliminating reactions that cannot carry flux (orphans not connected to the remainder of metabolic network) (details in Johnson *et al.*, 2017).

1.4 Integration of Gene Expression Data

A consensus assembly was gnerated using Trinity (version 2.2.0) (Grabherr *et al.*, 2011). Expression levels were calculated using RSEM (version 1.2.22) (Li and Dewey, 2011) and Bowtie (version 1.0.0) (Langmead *et al.*, 2010). Annotations (including GO categorization) were generated using Trinotate (version 3.0.0) (www.trinotate.github.io) within the Trinity pipeline (Grabherr *et al.*, 2011):

Total trinity transcripts: 178,982 Percent GC: 43.74

Statistics based on ALL transcript contigs

Contig N10: 402
Contig N20: 2010
Contig N30: 1507
Contig N40: 1171
Contig N50: 899
Median contig length: 389
Average contig: 646.45
Total assembled bases: 115,702,320

Statistics based on ONLY THE LONGEST ISOFORM per 'GENE'

Contig N10: 3713
Contig N20: 1969
Contig N30: 1480
Contig N40: 1146
Contig N50: 875
Median contig length: 384
Average contig: 635.23
Total assembled bases: 109,823,815

Scheme 1. *Flowchart outlining development of the Pintae_Epi and Pintae-Meso models.*

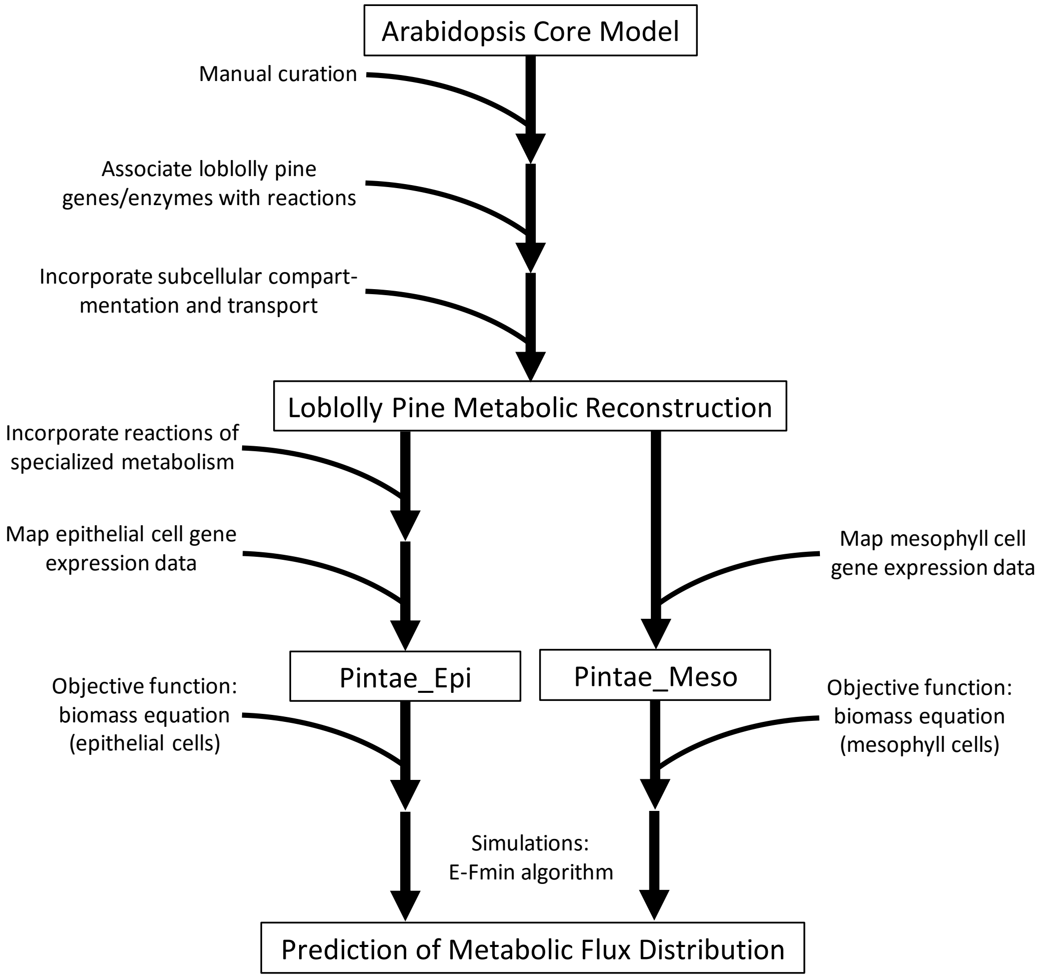

The contigs of the loblolly pine epithelial cell and mesophyll cell transcriptome assemblies were compared, using the Blastx algorithm, to the UniProt and TAIR sequences represented in the MetaCyc and AraCyc databases, respectively. Loblolly pine transcripts were associated with reactions from the Arabidopsis Core Model, AraCyc or MetaCyc (in this order of priority), based on global identity, and this information was transferred to our metabolic reconstructions. Reactions in the metabolic reconstruction with no associated transcripts in the appropriate data set were removed. These additional steps generated the metabolic models for loblolly pine epithelial cells (Pintae_Epi; 694 reactions) and mesophyll cells (Pintae_Meso; 722 reactions) (Scheme 1).

1.5 Determining resin duct volume

The oleoresin concentration had been determined for bulk needle tissue. For the Pintae_Epi model, it was important to calculate the production of oleoresin by individual resin ducts and their epithelial cells. We therefore determined the volume fraction of needles occupied by resin ducts. Details are described in the Materials and methods section of the main manuscript and a visual description is provided in Scheme 2.

Scheme 2. *Morphometric determination of fractional volumes of resin ducts.* ***A****, Use of the "Freehand" function of ImageJ to obtain cross-sectional areas of leaves.* ***B****, Tapering of leaves at the distal end.* ***C****, Modeling the shape of the leaves tip as elliptical cone.* ***D****, Use of the "Oval" function of ImageJ to obtain areas of resin ducts.* ***E****, Modeling the shape of a resin duct as cylinder.*

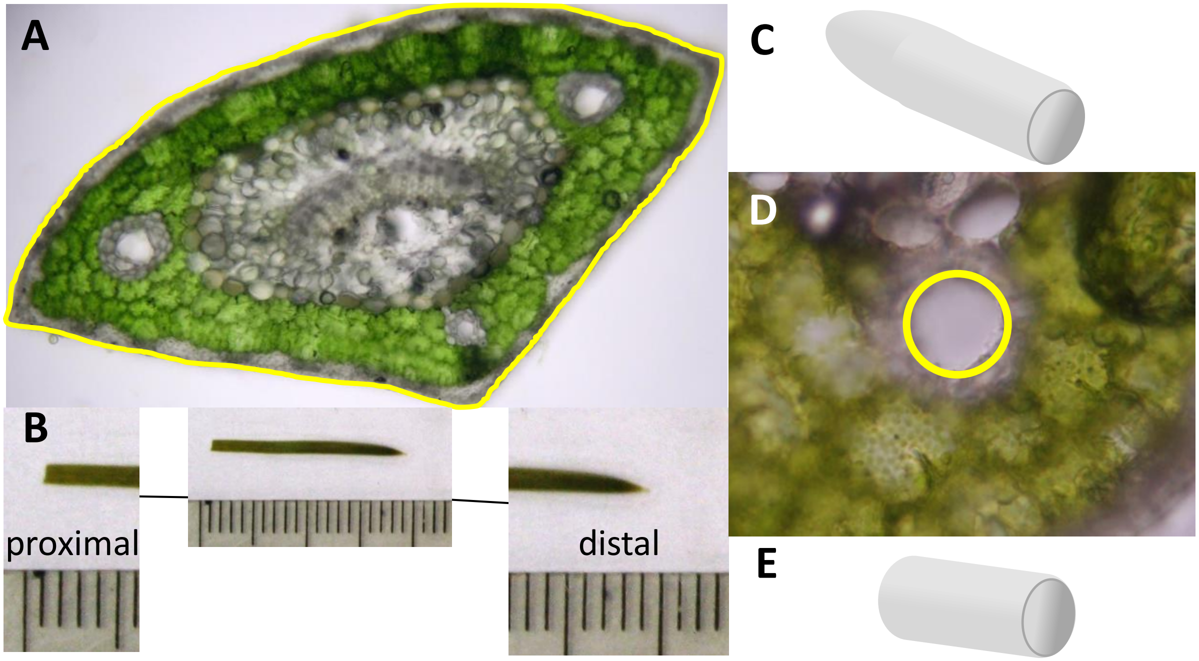

1.6 Application of Flux Minimization Principle

Gene expression values (as Transcripts per Kilobase Million or TPM) reflecting averages of three replicate transcriptome data sets obtained with isolated epithelial cells of resin ducts and mesophyll cells (NCBI Sequence Read Archive, accession number SRP126587) were mapped onto each reaction of the Pintae_Epi and Pintae_Meso models, respectively. In instances where multiple genes code for an enzyme and/or multiple enzymes are associated with a reaction, the cumulative TPM for each reaction was calculated. To computationally predict intracellular flux distribution, a slight modification of the Expression data-guided Flux Minimization (E-Fmin) algorithm was applied (Song *et al.*, 2014). The E-Fmin algorithm was chosen due its capability to analyze complex metabolic networks, and its ability to consistently predict flux distribution using a stoichiometric network and gene expression values for the enzymes involved in the network.

Supplementary References

Arnold A, Nikoloski Z. 2014. Bottom-up metabolic reconstruction of Arabidopsis and its application to determining the metabolic costs of enzyme production. Plant Physiol. **165,** 1380–1391.

Caspi, R., Billington R, Ferrer L, Foerster H, Fulcher CA, Keseler IM, Kothari A, Krummenacker M, Latendresse M, Mueller LA, Ong Q, Paley S, Subhraveti P, Weaver DS, Karp PD. 2016. The MetaCyc database of metabolic pathways and enzymes and the BioCyc collection of pathway/genome databases. Nucleic Acids Res. 44, D471-D480.

Hooper CM, Tanz SK, Castleden IR, Vacher MA, Small ID, Millar AH. 2014. SUBAcon: a consensus algorithm for unifying the subcellular localization data of the Arabidopsis proteome. Bioinformatics **30**, 3356–3364.

Johnson SR, Lange I, Srividya N, Lange BM. 2017. Bioenergetics of monoterpenoid essential oil biosynthesis in non-photosynthetic glandular trichomes. Plant Physiol. **175,** 681-695.

Lamesch P, Berardini TZ, Li D, Swarbreck D, Wilks C, Sasidharan R, Muller R, Dreher K, Alexander DL, Garcia-Hernandez M, Karthikeyan AS, Lee CH, Nelson WD, Ploetz L, Singh S, Wensel A, Huala E. 2012. The Arabidopsis Information Resource (TAIR): improved gene annotation and new tools. Nucleic Acids Res. **40,** D1202–D1210.

Song HS, Reifman J, Wallqvist A. 2014. Prediction of metabolic flux distribution from gene expression data based on the flux minimization principle. PLoS One **9,** e112524.
